# Understanding wellness in a context of harm: Gaza’s Pandemic mental health success and the Palestine Trauma Center

**DOI:** 10.1371/journal.pgph.0006290

**Published:** 2026-04-20

**Authors:** Rasha Bayoumi, Oliver Bones, Mohamad Altawil, Betelhem Wolde, Nora Parr

**Affiliations:** 1 School of Psychology, University of Birmingham Dubai, Dubai, United Arab Emirates; 2 School of Humanities, Social Sciences and Health, University of Wollongong Dubai, Dubai, United Arab Emirates; 3 Palestine Trauma Center, Hertford, Hertfordshire, United Kingdom; 4 School of English, Drama, and Creative Studies, University of Birmingham, Birmingham, United Kingdom; University of Pennsylvania Perelman School of Medicine, UNITED STATES OF AMERICA

## Abstract

Mental health interventions in contexts of ongoing violence are challenged with supporting psychological well-being in the absence of external safety or stability. In such contexts of harm, trauma is often persistent rather than time-limited, necessitating approaches that emphasize adaptation, an internal notion of safety, and resilience rather than symptom elimination alone. This mixed-methods study investigates changes in PTSD indicators associated with Tarkiz, a culturally adapted community-based PTSD intervention delivered in Gaza before and during the first wave of the COVID-19 pandemic. Quantitative data from 419 participants were analyzed to assess changes in trauma symptoms following participation in the intervention and to examine whether symptom change differed by time period of intervention administration (pre- vs. during COVID-19). A mixed design 2x2 ANOVA revealed a statistically significant decrease in mean trauma scores after the intervention in both time periods, with a Mann-Whitney test confirming a larger reduction in mean trauma scores for individuals who participated in the intervention during the COVID-19 period. Participant and practitioner interviews constituted the qualitative section, highlighting what was understood in the community as reasons for the perceived success of Tarkiz. This included the emphasizing of communal support, and tools to function meaningfully amid pervasive instability, such as projective externalization and creation of an internal safe space. Overall, these findings suggest that participation in Tarkiz was related to reductions in trauma symptoms potentially due to its understanding of an ongoing context of harm and focus on re-orienting individuals to address these harms as a community and in a continuous capacity. This study also offers insights for extended implementation of mental health interventions in Gaza and similarly constrained settings.

## 1 Introduction

Research on the impact of COVID-19 has pointed almost universally to its exacerbation of existing health and mental health conditions [1]. Since the outbreak of the pandemic in 2019, there has been a proliferation of research not only on how the pandemic complicated individual medical issues such as asthma, heart disease, and immunocompromised systems [1], but also larger social and structural conditions which intersect with race [2], gender [3], socioeconomic status [4], and culturally specific norms [5]. Globally, this research found that already vulnerable populations, such as residents of low and middle-income countries (LMICs) in the Global South and war-affected areas, were the most at risk for adverse mental health effects due to the pandemic. This work has also highlighted the limitations of existing mental health programs, which were by and large unable to make a meaningful difference in many LMICs and communities of color, due to a failure in developing culturally relevant alternatives to traditionally white and high-income countries (HIC)-focused treatments [6–8].

While the bearing of the social determinants of mental health was universally worsened by COVID-19, this was particularly the case in LMIC and war-affected regions [9]. For instance, while residents in HICs tend to have access to income support and employment options that allow them to work from home, a substantial portion of the LMIC population is engaged in informal labor, resulting in worse economic consequences of job loss [10,11]. Similarly, many LMICs and war-torn countries struggle with consistent access to clean water and other basic necessities, making it difficult to comply with strict lockdown measures imposed, resulting in violent crackdowns from law enforcement [12,13]. These systemic issues significantly compounded the mental health crisis experienced during COVID-19, such as increased anxiety and depression as a result of isolation and uncertainty. Given these at-risk communities, defaulting to existing treatments, which by and large fail to address these systematic issues in LMIC, poses serious problems. This calls for the development of more context-specific intervention strategies for these communities [10].

Given this context, this paper aims to investigate the gap in existing PTSD programs in vulnerable communities, particularly by spotlighting the multi-layered nature of harm in Gaza. Crucially, the study also introduces and investigates how the Palestine Trauma Center (PTC) and its PTSD treatment program, Tarkiz, address these limitations during the pandemic, arguing the importance of the program’s sustained cultural grounding in its treatment of a community experiencing compounding crises.

### 1.1 Defining ‘context of harm’ in terms of Mental Health

Historically, trauma has often been understood as a “lag, a snatch, in the experience of the traumatized that pulls them out of linear chronology” [14,15]. It is most classically the case in PTSD treatment that working ‘through’ or getting ‘over’ a traumatic incident imagines both that the incident is over, and that it was limited in scope [16]. As a result, PTSD-informed programs in the Global South have been criticized for failing to meet the cultural needs of its patients [17], additionally often embedding in them normative assumptions about race/class/other that exclude the experiences of communities in the Global South [18,19], and having a problematic understanding of ‘recovery’ [20]. While programs like Stress First Aid and Psychological First Aid are said to have been effective in the context of the pandemic (and were developed with mechanisms of some basic cultural adaptation) [21], they both make the assumption of the availability of a follow-up, which for many vulnerable communities may not be possible, as life after the pandemic is full of other layers of harm.

Consequently, The Palestine Trauma Center (PTC-UK, henceforth PTC) and their program Tarkiz (focusing) focus on this structural and continuous nature of trauma, and aim to provide individuals with the tools to build strong community networks and inner safe spaces when external stability and safety may not always be a reality. PTC and Tarkiz operate based on an experience of trauma as not merely opposite to Stonebridge [14]’s ‘lag’ analogy, but through a different structure of imagining entirely. Here, trauma *is* time, it is the experience of multiple, ongoing, intersecting, and multi-dimensional violences that in essence create the fabric of life experience.

COVID-19 in the Gaza Strip is an example of a context of harm. In delineating what we can of the types of harm that the PTC community exists within, the aim is to demonstrate the intersections, continuity, multiplicity, and many magnitudes of violences; because it is this that constitutes a context of harm. From colonialism, racism, economic depression, isolation, military targeting to interpersonal, government, and family violence—the types of violence matter less than their multiplicity. In this paper, we relay Gaza’s context of harm in two ways. First through what has become a typical evaluation of some of the measurable and quantifiable indicators of life quality, largely based on I/NGO monitoring. Second, we offer the case histories of three women who attended PTC sessions. How these histories were collected is detailed in the Methods section. While this concept of a context of harm is being illustrated through the experience of COVID-19 in Gaza, it is not specific to this context. Rather, we contend that this concept might provide a better starting place for mental health interventions for vulnerable communities more generally.

Harms in Gaza are continuous and intersecting. They have accumulated to such a degree that the World Health Organization (WHO) predicted the area would be “unlivable by 2020” [22]. This was based on evaluations of access to necessities like food, clean water, safe housing, and medical care. Gaza was nominally administered by Egypt until 1967 when it was occupied by Israel. While there was a formal withdrawal in 2005, the area remains under an Israeli military siege. Access to areas outside of Gaza is restricted by Israel and Egypt, along all land and sea borders. This has affected all aspects of life from trade to medical treatment; supplies entering Gaza are restricted as are the people seeking to exit. This has stifled both life of the flesh and the economy. According to its last published statistics in 2017, the UN Office for the Coordination of Humanitarian Affairs (UNOCHA) recorded a 46% unemployment rate, where 47% of households in Gaza suffer from moderate or severe food insecurity, 97% of piped water was unfit for human consumption—even while more than 70% of Gaza’s population receives some form of international aid, the bulk of which is food assistance [23]. These are just the systemic issues.

Harms in Gaza come in many different magnitudes, from the political-systemic to the cultural-systemic (domestic violence, gender and class discrimination, social stigmatizations) and event-based. There have been regular Israeli military operations carried out on the area over the last two decades, which by then were known as “mowing the lawn” [24]. These have seen tens of thousands of homes destroyed, thousands killed, even more injured, and the routine bombardment of infrastructure such as hospitals and electrical plants [23]. This compounds the structural harms. Added to this is an uncertain internal political context in which two Palestinian governments (a Hamas-run local authority and the Fatah-lead and internationally recognized Palestinian Authority) operate unevenly and with patchy coordination around public services [25]. This is the ‘baseline’ quantifiable version. Then came the pandemic.

During the pandemic’s first wave in 2020, the already largely fragmented healthcare system in Gaza struggled to contain the virus and efficiently rehabilitate those infected, as only 87 ventilator-equipped intensive care unit beds were available for a population of nearly 2 million [26]. Additionally, strict Israeli restrictions on movement continued to create barriers for Gazans to receive comprehensive treatment that was not available locally [27]. This, compounded with uncertainties about the novel virus, existing public health crises such as poverty and electricity and water shortages, as well as mass unemployment due to the lockdowns, contributed to an increase in the prevalence of depression, anxiety, and overall psychological distress among those living in Gaza [28]. Furthermore, the restrictions on gatherings proved to be detrimental to a population that has historically relied on social support in times of distress, especially at a time when Israeli military violence was reported to have increased, further escalating feelings of worry and tension [28]. In order to best address the psychological needs of populations such as Gaza’s, the intersection of multiple and overlapping harms must be tackled in the development of intervention programs.

This is demonstrated in this paper through the case of PTC and their program Tarkiz [Focusing]. Crucially, PTC’s mental health provision is not meant to normalize a problematic context of harm. In the case of Gaza in particular, and vulnerable communities in general, providing mental health work cannot change the harm that individuals have and will continue to experience. Nor does the program pathologize the individual. Instead, Tarkiz attempts to address the complex harms faced by its community. It defines success through the mitigation of locally adapted and verified PTSD indicators [29], and enabling both individuals and the communities they are a part of to thrive despite the status quo—and even the support to continue working against it. In the context of Palestine, this means mental health is a form of resistance and subtends the political work of finding an equitable way to practice good mental health in the face of occupation - and in the case of Gaza, of ongoing siege and bombardment [30].

Since its founding, PTC has developed intervention programs that work on a community level to empower families and individuals and cultivate personal and community-based skills that help combat stress and trauma. In addition to Tarkiz, PTC offers programs geared toward children, families, and teens. These include the Family Therapy Program, which takes the family as a unit of analysis while still focusing on individual mental health; the Psycho-Social Support Program, which tends to the social context within which psychological symptoms appear, and Community Wellness Focusing, which focuses on the irreducible role of the community in the practice of building resistance [31]. They also offer programming for children, including a Friday of Joy initiative, which brings psychosocial support and community-making into the streets; reclaiming spaces of violence, often where bombings or bombardments have created open spaces in the dense urban infrastructure. Friday programs, which include plays, minor circus acts, and pantomimes, work to destigmatize issues around mental health, and share wellness solutions with children and their families. As its services are free, and on a first-come first-serve basis, the center is constantly inundated with community members and referrals from the local Ministry of Health, schools, and other NGOs. And while it does ‘score’ individuals on an adapted PTSD score sheet, this does not determine whether or not anyone can access a program.

### 1.2 The Palestine Trauma Center in Gaza: Addressing a context of harm

PTC was founded in this aforementioned context of harm and has crafted a set of programs in an attempt to tackle its many different layers. The center is a small NGO operating in Nuzierat, a region in the Middle Area of the Gaza Strip. Its founding director Dr Mohamed Altawil, along with the governing board, is based in the UK, which is where the center does most of its fundraising operations and is registered as a charity. The organization has faced multiple challenges, both financial and other, and is built on a model meant to weather the challenges that life in Gaza entails (recognizing need, developing to scale, first come first served, free to access, reducing stigma).

As there is no consistent government infrastructure, and as internal political conflict has hampered efforts at providing already underfunded services [32], all of the center’s expenses are paid for by donations, in addition to occasional small sources of funding around research like the present study (This study was funded with a 5,000 GBP UKRI-IAA COVID Urgency grant through the University of Birmingham, 2021). This not only addresses the financial and political instability of the Gaza Strip, but also the reality of extreme poverty that the PTC community exists within. Free services reduce the financial barrier to mental health care, but as PTC sees it, also helps reduce the stigma associated with mental health services. Because of a shortage of trained psychiatrists, psychotherapists, and training procedures, most other NGOs, including PTC, offer services developed by professionals and run by local workers who receive appropriate training to facilitate the programs, often with backgrounds in social work and community organizing [33].

All of the services run for free, all of the time. Staff work closely with community members to identify times and spaces to run programs so that they can be accessed as safely and regularly as possible. Community focus and communal cooperation continued to drive the PTC’s programs as the first wave of the pandemic hit, with staff giving out PPE, hand sanitizer, and leaflets about how to protect oneself and family from the virus. They also released a series of animated videos in Arabic on their YouTube channel explaining what was known at the time about the virus. They then rapidly converted their most subscribed in-person program and made it available for online delivery, with group Zoom calls creating a novel space for members of the community to share their struggles and find solace in their fellow residents. The Tarkiz program was thus run nearly continuously through the first wave of the Pandemic.

### 1.3 Tarkiz: Translation and cultural adaptation of ‘Focusing’

“Focusing Intervention for Self-Caring & Psycho-Social Support” (Arabic: *al-tarkiiz li al-ra’aiia al-dhatiyya wa al-d’am al-nafsy*) or “Tarkiz,” is an adaptation and translation of a theory devised by the American philosopher Eugene Gendlin in the 1930s. Focusing puts emphasis on recognizing personal and individual responses to harm and understanding reactions to these [34]. Consequently, the program’s core philosophical ideology relies on avoiding the assessment of the individual as a closed unit of analysis, which had become a near dogma in American ego-psychology after World War II [35]. Instead, Tarkiz referrals are coached in the identification and externalization of harm, separating structures, events, and processes of harm and difficulty from the person who has experienced (and is experiencing) them. This allows individuals to identify and separate the causes of harm to imagine their inner life as something that is affected by but not constituted by grief or violence. Instead of relying on ‘safe spaces,’ which by and large do not exist in Gaza, Tarkiz asks participants to find strength in self. The self becomes the safe place, not only through identification of personal strength, but by connecting to others in the community. This comes through a focus on listening and acknowledging the feelings of others amidst the violence and disruption they may experience, highlighting Tarkiz as a program focusing on community-based agility in crisis. This is in direct opposition to the vast majority of traditional PTSD treatments that often isolate the individual ‘victim’ from their broader community and cultural context [36].

After being personally impressed by the Focusing technique, Altawil adapted and tested it with Arab students at Hertfordshire, went to Gaza in 2010 for the first training course, and has continued to fine-tune the program. This has largely been in response to client feedback and staff experiences, but research on the precise nature of the cultural adaptation is ongoing (See the Rights for Time network [37], for which work with the Palestine Trauma Center forms a case study in the re-definition of ‘protection’ responding to a UK government call for work from the humanities addressing the question. This work is funded by the Arts and Humanities Research Council.)

Having established the context and structure of the PTC and Tarkiz, the following section outlines the research methods utilized to assess mental health outcomes related to the program during the pandemic via the monitoring of PTSD indicators in participating individuals.

## 2 Methods

We developed a mixed-methods approach that worked with PTC’s existing systems, combining qualitative case studies with quantitative data from questionnaires. Qualitative information illuminated participants’ lived experiences in the context of Gaza’s overlapping harms, while the quantitative section provided statistical evidence of differences in trauma scores pre- and post-intervention.

### 2.1 Quantitative methods

For our quantitative section, we were able to compile data from questionnaires (assessment sheets) administered to 105 individuals who took part in Tarkiz before COVID-19, and 211 individuals who took part during the COVID-19 Pandemic. Baseline data was collected in April-July 2020 before the outbreak of COVID-19 in Gaza. The second set of data was recorded in October-December 2020, from when the first cases of COVID-19 were reported, as the first wave of the spread of the virus set in, and lockdown measures were imposed. Both questionnaire data and interviews were first accessed retrospectively by the authors for the purposes of this study on April 21^st^, 2021. This data is based on the intake and exit interviews and assessment sheets that are part of the basic operating structure of the PTC.

The forms are based on an adaptation of the DSM-IV criteria, and measure the following indicators:

a) Persistent recollection of harm, manifested in difficulty sleeping, nightmares, sound-related remembering, or physical pain;
b) Avoiding things that remind one of harm in conversation, physical places, objects, people;
c) Mood status including evaluations for “desire for living,” “appreciating value in life,” pessimism, fear of the future, rapid mood changes, lack of safety;
d) Social balance including acting inappropriately with family/at work/school;
e) Ability to focus;
f) Professional functioning including ability to complete necessary tasks;
g) Maintain relationships with friends and family, and participation in social events.

This information was used for two reasons: first, as noted above, it was available and meant no additional burden of data collection for staff. Second, the questions and metrics were developed locally and were not a superimposed Global North assessment. The forms assess harm as it is perceived by the PTC community.

A mixed-design 2x2 ANOVA was used to examine the effects of Tarkiz on trauma scores within-subjects (pre- and post-intervention) and between-subjects (intervention received before or during COVID). Additionally, a Mann-Whitney test was used to analyze the differences in changes in trauma scores between participants who received the intervention before versus during COVID-19. A chi-square test of independence was also used to examine whether PTSD diagnostic status following intervention (whether participants were ‘free’ or ‘not free’ of PTSD symptoms) was associated with the time period in which the intervention was delivered (before vs during COVID-19).

As participant scores were obtained retrospectively, an a priori power analysis was not conducted. Instead, a sensitivity analysis was conducted to determine the minimum detectable effect size given the sample obtained (N = 316). With α = 0.05, and a two-sided test, this study had 80% power to detect effects of at least f = 0.19 in a 2x2 ANOVA.

IBM SPSS Statistics 29 was used for all quantitative analyses.

### 2.2 Qualitative methods

For the qualitative section, staff took detailed notes and carried out informal interviews with three Tarkiz clients during both the entry and exit interviews amid the first wave of the pandemic. Staff volunteered to carry out the interviews, and when, who, and how were left up to the staff, so as to place as little pressure on them and the PTC’s operations as possible. Three different staff members interviewed the three different participants who agreed to have their assessment shared (anonymously) at three different moments during the first wave of COVID-19 cases in Gaza. The data gathered was of three women, all married (or widowed) and in mothering roles. As is usually the case with qualitative interviews, the aim was not to offer fully generalizable data [38,39], rather, it was to ethically capture the subjective experience of the participants, to offer some details of a human experience of the COVID-19 Pandemic, to understand the effects of the experience on daily life, and mental health. The qualitative analysis conducted is further detailed in a previous study [40].

Parallel to these data sets, sessions were held with staff to gain background and insight into the rapid change in program delivery. Sessions constituted informal and non-structured interviews. These discussions will not be reported on in full, but rather the content is used to elucidate the participant interviews. Further details on how Tarkiz was culturally adapted and received by the community are discussed in a previous study [40].

### 2.3 Ethics

This study received approval from the University of Birmingham ethics committee (Ethical Review ERN_20-1308A). Relevant local authorities in Gaza reviewed the approval and confirmed that a separate ethics submission was not required for this project. The project was conceived and implemented in partnership with Dr. Mohamed Altawil, the founder of the Palestine Trauma Center, as part of a UK government funded research network (Rights for Time/ R4T) [37]. Involved from the project’s inception, PTC and four other stakeholders from the global south were also key in developing the project’s ethical protocols, with which this work is in line. Participant information was anonymized at the source (PTC), including names, addresses, phone and ID numbers, and directly identifying information, and authors had no access to this data. Additionally, participants that were interviewed were assigned pseudonyms for the purposes of this study.

## 3 Results

### 3.1 Quantitative results

In this section, we present quantitative data evaluating Tarkiz’s impact on overall trauma symptoms, PTSD diagnostic status, and specific PTSD domains during the first COVID 19 pandemic lockdown. These results are compared to outcomes recorded in the months immediately prior to the appearance of the first cases of COVID-19 in Gaza.

#### 3.1.1 Overall trauma symptoms.

**Fig 1:**
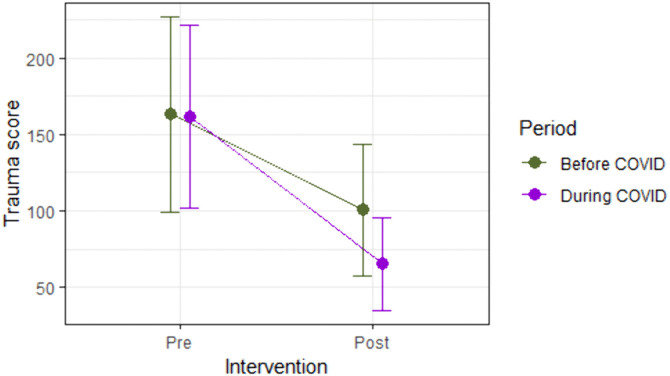
Trauma scores pre- and post-intervention, before and during COVID. Error bars indicate 1 standard deviation from the mean trauma score.

As displayed in Fig 1, mean trauma scores decreased from pre- to post-intervention in cohorts participating both pre-COVID (n = 105) and during the first wave of the pandemic (n = 211), with a steeper decline observed in the latter cohort. In line with this, the 2x2 ANOVA revealed that time of testing (pre- or post-intervention) had a large effect on trauma score (*F*(1, 314) = 874.85, *p* < 0.001, *η*^*2*^_*p*_ = 0.74), indicating a statistically significant reduction in trauma scores from pre- to post-intervention, both before and during the pandemic. Time period (before or during COVID) also had a moderate effect (*F*(1, 314) = 2118.02, p < 0.001, *η*^*2*^_*p*_ = 0.04) on trauma scores, and, importantly, the effect of time of testing and of time period Interacted (*F*(1, 314) = 40.04, *p* < 0.01, *η*^*2*^_*p*_ = 0.11). This indicates that the magnitude of reduction in PTSD symptoms differed by time period.

**Fig 2:**
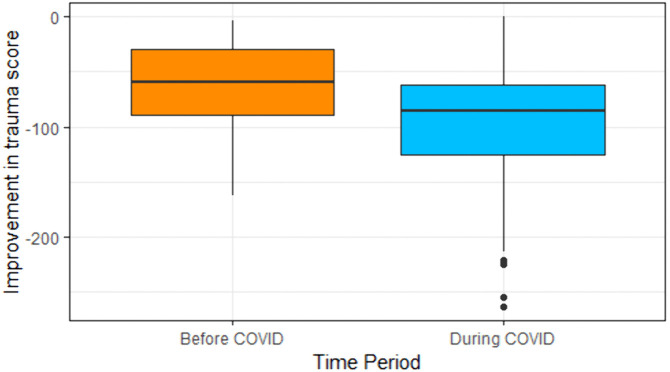
Improvement of trauma scores before and during COVID. *Values represent change in trauma score calculated as post-intervention minus pre-intervention, with more negative values indicating greater symptom improvement.

To explore this interaction further, changes in trauma score (post-intervention – pre-intervention) were similarly calculated for participants who received the treatment before COVID and during the first wave of the pandemic. As supported in Fig 2, results from Mann-Whitney analysis reveal that changes in trauma scores were significantly greater during COVID (median change in trauma score = -86) than before COVID (median change in trauma score = -59; *U* = 6469, *Z* = -6.02, *p* < 0.001), with a medium-sized effect of *r* = 0.34.

#### 3.1.2 PTSD diagnostic status.

In addition to symptom reduction, we assessed the proportion of participants that no longer fulfilled PTSD criteria following the Tarkiz intervention. Table 1 displays the number of clients who were evaluated as ‘free’ or ‘not free’ from PTSD following their participation in Tarkiz, both before and during COVID. These categories are based on measures that were locally adapted based on the DSM-IV criteria for PTSD and are not a formal diagnosis. While a greater proportion of clients were free of PTSD after partaking in the intervention during COVID (78%) as opposed to before (63%), this relationship between time period (before COVID; during COVID) and PTSD status after intervention (free; not free) was not statistically significant, χ²(1) = 6.21, *p* = 0.13.

**Table 1:** Frequencies of PTSD status post-intervention before and during COVID.

Time Period	PTSD status post-intervention	
	Free	Not-free
**Before COVID**	57	33
**During COVID**	136	39

#### 3.1.3 Specific symptom domains.

Lastly, we assessed the difference in individual symptom indicators before and after Tarkiz. Analysis revealed that Tarkiz mitigated two mental health indicators that saw a sharp rise for clients during the Pandemic: inability to focus, and inability to sleep. Table 2 shows a random sampling of clients reporting sleep difficulties before and after the Tarkiz program, with difficulty ranked 1 (low) and 10 (high). Scores were once again compared between participants that received treatment before and after the first wave of the pandemic. Results show that both baseline numbers and improvements were higher during the pandemic. Table 3 similarly shows a random sampling of clients reporting difficulty completing tasks before and after the Tarkiz program, with scores compared between participants partaking in the program before versus during the pandemic. While reported numbers here remain fairly consistent pre- and post-pandemic, improvement is increased in the cohort that participated in the program during the pandemic.

**Table 2:** Comparison between the reporting and resolution of ‘sleeping difficulties’ before and during the outbreak of COVID-19.

*Before* COVID-19		*During* COVID-19	
Pre-Assessment	Post-Assessment	Pre-Assessment	Post-Assessment
9	4	9	2
10	7	9	2
8	7	10	3
7	3	10	1
7	4	10	3
7	5	10	2
**Avg = 8**	**Avg = 5**	**Avg = 9.6**	**Avg = 2.1**

**Table 3:** Comparison between the reporting and resolution of ‘difficulty in continuously doing things’ before and during the outbreak of COVID-19.

*Before* COVID-19		*During* COVID-19	
Pre-Assessment	Post-Assessment	Pre-Assessment	Post-Assessment
6	4	10	2
10	6	3	0
9	6	6	0
8	6	8	2
7	2	10	3
7	4	8	2
**Avg = 7.8**	**Avg = 4.6**	**Avg = 7.5**	**Avg = 1.5**

### 3.2 Qualitative insights

The quantitative data reveals that participants experienced a significant reduction in trauma symptoms after participating in Tarkiz during the COVID-19 pandemic, but does not provide insights on why this may be the case. As the indicators for trauma are also so often different from what clients described as their main complaint, patients in their qualitative assessments did not describe success of the program through, for example, the regained ability to sleep or the disappearance of nightmares. Indeed, such a 1:1 correspondence seems better suited to a PTSD-informed approach that sees indicators as a response to a single experience. When participants and practitioners spoke of the achievements of the program, success was defined as that which impacted—either at its core or from different approaches—the way that individuals interacted with their context of harm.

Before discussing the ways in which the program impacted them, we first provide context by illuminating the reality of intersecting harms in the lives of three women; Amal, Fatima, and Muna, from before they began participating in Tarkiz:

**Amal**

Amal is a 28-year-old married woman and stepmother whose marital home was destroyed by an Israeli military airstrike during what was termed ‘Operation Protective Edge’ in the summer of 2014. Her parents both died of treatable kidney conditions within a year of each other before the bombardment, attesting to the crumbling infrastructure of Gaza’s healthcare industry. Amal cannot have children of her own. She, her husband, and her stepdaughter live in extreme poverty in an area regularly targeted by airstrikes. Amal was referred to PTC in October 2020. She had stopped leaving the house and attending family functions and refused to participate in her community. Her in-person sessions were cancelled when the virus broke out in the Gaza Strip. At this point, her husband lost his job. During the program, Amal contracted the coronavirus, suffered moderate to severe symptoms, and was stricken by an intense fear that she would infect her remaining family members. She continued the Tarkiz program online during her isolation.

Perhaps surprising given all these very obvious harms, is how Amal described herself and her condition in a pre-assessment interview (done before contracting COVID-19). Asked about the most difficult thing she was dealing with, she spoke of the loss of her father: “I am unable to forget my father’s death; the memories of the funeral remain with me. The pictures of medicines and bleeding are still images firmly in front of my eyes.” She described nightmares and physical pain that manifested in her feet, and a desire to avoid locations that reminded her of her father’s death. As the family patriarch, his death had marked the end of a time when Amal felt protected. So, while Amal had faced innumerable hardships since her father’s 2013 passing, in her own narrative, it was this moment that would best illustrate her mental state. In fact, all of the other related issues that Amal described to the PTC staff member relate directly to the absence of the security of a father and patriarch, a figure meant to protect the family.

Amal recalled feeling safe at a neighbor’s house during the bombardment of 2014 after her home was destroyed: “We took shelter in a neighbor’s house and remained living there in a state of disruption, insecurity, but also safety after the bombing of our house.” In speaking to her interviewer, however, this insecurity only reminded Amal of the loss of her parents, a sort of back-up unit of support whose loss she felt keenly. In her words, she would avoid all of the things that made her feel precarious: old neighborhoods, homes, cemeteries, and even extended family gatherings. Amal also brought up her infertility. She feels in a sense unable to ‘create’ her own family (the primary social care network) and is painfully reminded of this when she sees relatives with children. She looks back on her life with her parents—before their deaths, her marriage and discovered infertility, and also the string of bombardments on the Gaza Strip—as a time and environment of “love and respect.”

The practitioners at PTC assessed Amal as suffering from an “overwhelming accumulation of psychological and physical stress symptoms.” As Amal described it in her post-program interview: “Blackness had begun to haunt my thoughts and feelings and everything that beats with joy (c. 2014). When I started to feel a bit better [taking part in the program], I was infected with Coronavirus. My relationship with my family became stressful and full of fear.” The Pandemic further imperiled the only unit of support (the family) that Amal felt she had. Not only did she contract the virus and felt she was putting her husband and stepdaughter’s health at risk but she feared she would lose them as well.

**Fatima**

Fatima is a 56-year-old mother of six. Her youngest son died of acute pneumonia in 2009 after her attempts to find medical attention failed; hospitals were full, doctors had insufficient medicine, and she could not afford private fees. Her father was killed by a strike during an Israeli military bombardment that landed on the family home in 2014. The home was destroyed. Her sons (aged 25 and 35) are both unemployed. Fatima suffers from a cartilage disorder, which affects her neck and back. The family can no longer afford her medicine and are in debt. Fatima suffers from insomnia and nightmares. Returning to areas that were targeted for bombardment makes her nervous (this is where her family lives), as does being with her family all together. They had all been in the same building when it was hit in 2014, believing it to be the safest location. She felt persistent guilt about the death of her youngest son and feared losing her remaining children. Then COVID-19 struck, and Fatima felt certain all her family members would die.

In her first assessment, Fatima spoke at length about the death of her youngest son and her inability to protect him. This, she said, is what she has nightmares about most often. Of his illness, she narrates: “I was in pain and screaming inside every moment he felt chest pain when coughing or couldn’t breathe properly.” She went in search of help, “To save his life, I started knocking on hospital doors – hospital after hospital. There was no response. The hospitals were too crowded and had no supplies. He got worse and worse.” As she was desperately seeking medical support, trying to keep her son comfortable: “I went to check on him and he didn’t answer.” Her efforts at protecting her son, through her own ministrations or by seeking medical help, all failed. The lack of medicine in the Gaza Strip ensured this, and Fatima was powerless against the systems and structures that created the shortage despite her efforts.

Another experience that underscored Fatima’s sense of powerlessness was the 2014 Israeli military strikes on the Gaza Strip. Fatima was doing everything in her control to keep her family safe amid the strikes. As had become common, the residents of the apartment block where Fatima’s family lived were warned by the Israeli military of an impending strike. This can happen through phone calls, dropping of leaflets, or what has become known as ‘roof knocking,’ where small arms are dropped on a building as a ‘warning’ that a strike is coming [41]. Not all warnings are followed by strikes. The family decided to leave the building and take shelter with her husband’s family, whose home was nearby. They left quickly, as Fatima narrated: “My daughters and sisters and I sat through the night in the safest room of the house, while the intense bombardment went on. My husband sat with the men in a room next door. Suddenly a bunch of rocket shells crashed down on the next room. In the dense cloud of dust, I screamed and I screamed for my kids.” The children were fine, but her husband was killed. She continued, “with him I lost the safety I could feel. I was in a sea of pain.” She had done everything right but was again powerless to keep her family safe. She feared sudden death and was terrified of having her family all in one place. She told the PTC worker that she welcomed nightmares so she could remember her lost family members; her dreams were the only place she felt she had power.

**Muna**

Muna’s neighborhood is subject to repeated Israeli military strikes—it suffered damage in all three of the last large-scale incursions. She is constantly worried by the sounds of airplanes overhead. Her father was killed by an airstrike. When the pandemic hit, her husband lost his job. The family was already living below the poverty line. She feared infection and later contracted COVID-19. She constantly weeps over the death of her father, avoids going outside, has difficulty sleeping because of physical pain, and fears her own sudden death.

Muna’s experience of what are in essence the same harms as those detailed by Amal and Fatima boiled down to nothing less than total despair. In her narrative of her ‘condition’ with a PTC staff member, Muna focused less on the details of particular events or experiences, and more on the weight of their accumulation and how she felt beneath it. Like the other women, Muna is poor, she lives in an area where airstrikes are common and expected at any point in the year. She has also lost family members (her father) to aerial bombardment and feared a coronavirus infection. When asked to explain why she had come to the center, or what was most troubling her, she did not identify an external cause. She relayed, instead, being totally overwhelmed, and pulled out only the most recent experience as an individual ‘event’:

I imagine that death is near me. I lose the will to do any of my tasks in the family from fear. This fear keeps increasing in me and I have no energy. The crisis of the Corona pandemic puts fear upon fear: my husband lost his simple livelihood, we have almost no money and we lived in fear of infection. Then I became infected.

Muna’s concern with COVID-19 is that it’s yet another layer she can do nothing about, a “fear upon fear” over which she has no agency or control. As a PTC staff member commented in her notes, “She avoids talking about the trauma experienced and avoids visiting places that remind her of traumatic events.” There are so many of them in Muna’s history that she just doesn’t go anywhere or engage with anyone. As Muna tells her story, loss reads as an all-encompassing blanket. When pressed, she identifies, like Amal, the loss of her father as the ‘start’ of her despair, but the loss quickly folds into a larger tangle.

These are not easy case histories to read. It is, perhaps, easier to imagine a ‘normal’ life within which terrible things happen. Such a framework does nothing to understand the world that these women inhabit, let alone assessing their mental health, or further seeking to assist. However, it is in this context that the Palestine Trauma Center was formed and founded. It has been built to withstand the harms in which it operates, as well as assess and address them alongside its community.

Consequently, building from the data and the three case histories, as well as discussions with PTC staff members, three key elements of the program came up repeatedly. The first is constructing a unique sense of safety amidst a context of harm. This is an element of Tarkiz that has been adapted over the years and addresses the issue of the impossibility of an empirical sense of safety, as an external one simply does not exist. Here, the development of the program in a long-term context of harm was able to absorb the new layer of crisis that the pandemic represented. While a context of harm is nothing new to Gaza, the isolation brought on by the pandemic lockdowns took away crucial arenas of social support for the communities that PTC serves. The deployment of Tarkiz online fostered two new networks of social support that Amal, Fatima, and Muna each commented on, and which practitioners noted as supporting positive outcomes in participants. This was first, perhaps predictably, the online community of Tarkiz participants, but second, sharing what was learned during the sessions with family in shared isolation created new networks of support at home. Finally, in deploying Tarkiz online, PTC taught participants new digital skills, which created a sense of personal empowerment and growth during what was otherwise felt as stagnation or decline.

The sections below tackle each of these takeaways in turn. While this data is, as stated in the introduction, based on a small sample, it lends key indicators to the reasons for the success of the program during the pandemic. This supports the delivery of mental health programs that are adapted to contexts of complex and ongoing harm. In drawing out the particulars of the program’s success, what we see is its flexibility in tackling the experience of novel harms that are intricately tied to existing conditions. We attribute this flexibility as a trait generated through the program’s flipped focus. Rather than pathologizing the individual as suffering from poor mental health, the program postulates that it is the ‘situation that is sick,’ and gives individuals the tools to fortify themselves and their communities to navigate a violent context.

#### 3.2.1 Redefining safety.

Nothing about Amal, Fatima, or Muna’s lives can be made objectively safer through the intervention. However, Tarkiz does not necessitate safety as a prerequisite for healing. Instead, it innovates an approach to safety that is subjectively cultivated by the individual even within dangerous conditions. This is perhaps better termed security [42,43].

Tarkiz asks participants to imagine their own personal feeling of security — specific moments, memories, individuals, beliefs or experiences from their own lives that they can identify. This ‘safe space’ is a memory that makes individuals feel secure and creates an inner feeling of calm, security or safety. This helps remind individuals that life does contain elements of connection and experience that can make them feel safe. This struck a chord for Amal, who reflected, “The Safe Place session was very special. It helped me to remember the beautiful things in life, including my good husband, as the pain had blinded my eyes.” And for Muna, who told her PTC facilitator that the safe space:

Sessions were like flashes of light flowing into the dark cracks of pain. They brought many flashes of reassurance: my father’s beautiful memories, his pictures, his admirable life, my family and my children, my daughters, the Qur’anic verses about love, joy, hope; my faith in God, my belief that the next life is beautiful. All this created my Safe Place and gave me balance in this session. This really helped me a lot when I was infected with the Coronavirus.

For Fatima, this was even more transformative, allowing her to revisit things that were devastating and remember them as/when they were good. As she said, “In the Safe Place session, I imagined my dead son and my husband and all the sweet memories and felt thankful to God for His ability to satisfy and reassure the believer. Praise be to God!”

In essence, Tarkiz works to re-position participants vis-à-vis their experiences (the harms they endure) and the way they are experienced. Amal noted this as she reflected on one of the middle sessions of the program, the “sympathy session” where participants imagine that a stuffed animal or doll is the entity that has experienced their own harms. As Amal narrates, “I imagined myself as a doll, sad and lonely, who saw her parents in the shroud of death. This aroused some painful feelings, but in a relaxed way, so that I began to watch my condition with sympathy and feel I could start over.”

#### 3.2.2 Supportive communities beyond isolation.

Isolation was a huge factor in the deteriorated mental health of many during the pandemic. The appearance of the first cases of COVID-19 in Gaza brought sweeping changes in daily social patterns and saw many confined to their homes and cut off from larger family networks of support. People were now cut off from the rest of Palestine, from the global community, and also from each other—with no sense of when a respite might come. Further, rather than a clear adversary or a known set of military tactics, the source of fear, and the thing that people were isolating from was diffuse and unknown. The shift to the online provision of Tarkiz allowed continuity and establishment of community through co-participants.

In her post-assessment interview, Amal reflected on the elements of the program she found particularly useful. During the program, which during the pandemic was delivered in a group setting, Amal related: “I talked with others about my experiences. Their experiences were also difficult, and I felt that suffering in life may be a common factor for everyone. In the sessions we learned to empathize with each other’s suffering,” and in listening to each other created a sense of community and solidarity. When in-person group sessions went online due to the pandemic, Amal was initially skeptical. But because of the relationships she had forged with the other women participating, she learned how to access the online session and participated. Even when she suffered from COVID-19 symptoms and lost her voice, Amal listened to her peers. Likewise, for Muna, listening became crucial. As she reflected, “It was very important that I shared my experience. Hearing about the painful experiences of others was hard, but also inspiring. This flow between us and the concentration skills that were developed felt like the dressing of severe wounds.” For both, the program was in part effective because it did not individualize the problem, but rather showed it was collective.

Not only did this create bonds between Amal and the other participants in the program, but the lessons in connection were taken outside the group and into the home. Amal reported that listening and connecting with her Tarkiz cohort also gave her a fresh perspective on family relationships. “Later,” she told a PTC staffer, “I started sharing these skills with him [my husband], to start a new page with joy.” She understood through listening that her husband was also subject to the conditions that she faced. Rather than seeing him as an adversary, she began to explain elements of the Tarkiz program to him, so that they too shared in the listening and connecting exercises. This, she commented, allowed her to remember he was a kind and supportive person in her life; her new skills also allowed her to re-see value in the relationships she did have. The same went for Muna, who recalled that she: “told them [her children] about a precious skill called Empathy Exercise. I told them of the need for acceptance of everything that goes on inside us. I wanted them to understand that this was the key to breaking the ice and connecting with ourselves.”

#### 3.2.3 New forms of empowerment (digital).

All of the women in the case studies, and each of the staff members, spoke at length about the digital empowerment that was a novel outcome of the Tarkiz transition to an online program. The new skill, anecdotally, seems to have reinforced much of the work already achieved by the Tarkiz program. Access to Skype/Zoom and the online resources provided by PTC opened up a world of connection and lesson-amplification. PTC, in addition to running courses online, transitioned other community-oriented programs there as well, (those aimed at children, information on COVID-19 safety precautions, and general mental health awareness programs) becoming available to the program participants as they needed to navigate the resources in order to access the Tarkiz manual. In the short term, this empowerment affected no one more than Fatima. Because the digital media work meant that her confidence in using online resources increased, Fatima insisted on completing her university studies through e-learning. Other participants reported to PTC staff members that they were able to connect with loved ones and relatives beyond their household thanks to the new IT skills, and all reported a general increased confidence with technology (PTC is working on loan-laptops/tablets/wifi so that families without smartphones/wifi/computers can also participate). Clients reported being pleased with the new skill.

For Fatima, her own use of the online technology also encouraged interest from her children, partly because their mother was using technology she had not before, and partly because the conversations and interactions were taking place in the home. Once they took an interest, Fatima said she found “a new role teaching the children to cope with fear, dread, and anxiety,” while her facilitator commented that the family now “shared relaxation activities and drawing exercises for creating Safe Space, which distracted from worries about COVID.” Family connection was further amplified as PTC staff members transformed their Tarkiz teaching manuals into user booklets during the shift of the intervention to online spaces. Participants reported showing their family members the manuals and activities they were participating in, allowing for them to be replicated in the community.

For PTC’s directors, observations were that the “format complied with the principle of multi-sensory and psychoeducational learning,” ultimately enhancing and giving new resources to participants for self-directing. As Altawil reflected: “Focusing aims to be a client-directed process, not therapist-directed. Having the therapist distanced online and the client participating in the session while in the familiar home environment increased their autonomy and willingness to assume responsibility for the process.” He added, “Sometimes, the length of sessions themselves became more flexible and even more client-directed because of occurring in homes.” The flexibility of digital program development thus offered many unexpected benefits.

## 4 Discussion

This study investigated whether PTC’s Tarkiz intervention was associated with a reduction in PTSD symptoms in participants in Gaza within a context of harm characterized by continuous and overlapping violence, before and during the first wave of the COVID-19 pandemic.

Consistent with previous literature, preliminary results revealed an increase in baseline indicators of PTSD during the pandemic compared to immediately before. This may be attributed to several reasons, including the unpredictability of disease infection and its consequences, loss of loved ones and a lack of in-person social support, the latter of which is a staple in communal cultures such as that in Palestine and much of the Global South [28,44]. Additionally, majority of the Gazan population’s engagement in informal and/or manual labor meant that there were little to no opportunities for remote or alternative work, potentially causing job loss to be another significant factor in the increase in overall distress during the pandemic [10,11].

Results also revealed a significant reduction in PTSD symptoms after participation in the Tarkiz program both for individuals that received treatment before and during the first wave of COVID-19. This may be due to a number of factors related to the core tenants of the program. Firstly, Tarkiz’s reframing of the community’s context of harm meant that individuals did not have to navigate treatment on the basis that there was a clear start and end to their traumatic experiences, which has historically been foundational in PTSD treatment [14–16]. Instead, Tarkiz’s acknowledgement of the continuous and overlapping harms in Gaza meant that participants were able to re-orient themselves to address trauma as an ongoing phenomenon embedded within a broader context of harm [40]. This is crucial as the development of realistic expectations for treatment has often yielded positive results across a number of mental health interventions [45,46].

Additionally, Tarkiz’s recognition of the diminishing number of objectively safe spaces in Gaza due to continued Israeli occupation and bombardment is a foundational ethos in the development of the program [30]. Consequently, the intervention encouraged participants to develop a personal sense of security based on memories, people, places, etc. that have given them comfort in the past or those that soothe them through imagination. Guided imagery and safe place visualization have previously yielded positive outcomes in interventions in Gaza, South Africa, and among predominantly Middle Eastern and sub-Saharan African refugees in Denmark. Participants taking part in these programs similarly reported developing feelings of peace and security through these methods [47–49]. The self-guided nature of this strategy makes it feasible, especially when in-person treatment is not available, which is often the case in war-afflicted and resource-limited contexts [49].

Another aspect of the program that may have made it particularly effective is its addressal of feelings of helplessness and overwhelm, which are common in contexts of pervasive trauma and harm [50,51]. Particularly, the projective externalization participants were encouraged to participate in, where they attributed their traumatic experiences and emotions to a doll, allowed for the creation of aesthetic distance between participants and their traumatic experiences. This has been shown in previous literature as aiding the sensory processing of traumatic events and providing an opportunity for containment when these sensations may otherwise feel pervasive [52]. This externalization also allows individuals to observe events with more objectivity and regain a sense of control over their lives [52,53].

An additional key element of Tarkiz is its focus on the community as a unit of healing. Despite collectivism being a core element of Palestinian culture, stigma surrounding mental health still pervades much of the Global South, including Gaza [28,54]. As a result, communal interventions such as Tarkiz often allow participants to listen to each other with empathy, find solace in shared experiences and develop tools to support each other emotionally [40], playing a role in destigmatizing mental health treatment, improving its accessibility and addressing the community’s needs in line with its culture. This in turn can foster psychological and communal resilience in the face of ongoing and compounding harm.

Results also highlighted a significantly greater reduction in PTSD symptoms post intervention for individuals who participated during the first wave of the pandemic compared to before. While it may appear as though the pandemic had a protective effect on the community’s mental health, further exploration suggests that the greater reduction of symptoms during this time may have been due to the additional layer of harm in Gaza expanding the feeling of shared communal crisis and reducing stigma in help seeking behaviors [55]. Additionally, the expansion of Tarkiz’s delivery to an online modality meant that the intervention was more accessible and participants had the opportunity to attend sessions more consistently. As a result, the strategies taught through Tarkiz were embedded into many participants’ daily lives, even being shared with relatives in many cases. This can help strengthen the family as a feasible unit of support during volatile times, which can be crucial in fostering psychosocial resilience in instances of war and crisis [40,56].

The delivery of Tarkiz online also meant that participants gained skills and knowledge in an area previously unfamiliar to them. Their usage of technology therefore may have boosted their feelings of control, confidence, and empowerment during the pandemic [40]. This implies that the greater reduction in PTSD symptoms during the pandemic also reflects the alignment of Tarkiz with intensified communal disruption by expanding its accessibility and prioritizing the relevancy of its content and approach to the ongoing context.

### 4.1 Recommendations for implementation and future research

This study suggests that a culturally sensitive trauma intervention such as Tarkiz is associated with positive outcomes in the community that it serves. The construction and delivery of the program may be used as a reference for the development of other culturally adapted mental health interventions in similar contexts in the Global South, particularly in conflict-afflicted areas such as Gaza.

Firstly, it is vital that mental healthcare providers employ an approach that understands both trauma and recovery in these contexts as consistent processes, instead of being contained within a specific timeframe. Interventions should emphasize the cultivation of resilience, defined not as the lack of distress, but as the capacity to connect, adapt and function meaningfully in the face of persistent instability. This in turn allows providers to prioritize helping individuals develop a sense of internal security based on positive memories and feelings that can be accessed amidst external harm [47–49].

Treatment providers may also consider investigating the reasons that people are seeking support as a community, whether that be fear of places, trauma from strikes, sexual violence, etc. Tailoring coping mechanisms to these needs, such as the Safe Place strategy used in Tarkiz, can address symptoms effectively. In line with this, it can be useful to expand interventions to include group sessions with several members of the community, which many participants in previous Global South interventions have stated they prefer [40]. Relating and empathizing with others in similar situations often provides comfort and can help individuals develop the listening skills needed to aid each other when professional help is unavailable [40,57].

Additionally, it is important to structure group sessions in line with cultural preferences and norms; for example, Tarkiz allowed individuals to participate in sessions grouped by gender, allowing participants to feel more comfortable and share common experiences more openly. Various other cultures may have social norms related to religion, age, community roles, etc. which may be considered when implementing communal sessions [58].

This is more sensitively implemented when local workers are trained to deliver and monitor sessions instead of exclusively foreign healthcare providers in Global South and LMIC contexts [58]. This is also important as it maintains a level of accessibility to care when foreign aid is not possible. Making interventions available online is also crucial in expanding its availability, especially in contexts such as Gaza where in-person treatment centers may not be stable or safe to attend [59].

### 4.2 Strengths and limitations

This study has multiple strengths. Firstly, it provides a novel assessment of a culturally sensitive PTSD treatment in Gaza amidst the COVID-19 pandemic, undertaken collaboratively with the PTC. By using mixed-methods data, the study provides both scope and detail in comprehending the program’s impact. Crucially, the quantitative scales were developed and adapted locally though continuous monitoring of ongoing issues in Gaza and its inhabitants rather than imposed from Global North frameworks, ensuring that trauma indicators were appropriate within the social and cultural context of the territory. Concurrently, the qualitative section illuminated how participants made sense of rapid change within their broader ongoing context of harm. Additionally, data for the study was entirely collected through non-extractive methods; the study utilized data that staff had already collected as part of the PTC’s routine evaluation of its programs and participants, minimizing burden on frontline workers during compounding crises. This helped produce evidence that is ethical in its foundation.

However, this study is not without its limitations. While significant improvement in trauma symptoms was observed, there is a possibility of confounding influences from participants’ broader sociopolitical contexts. Secondly, although the quantitative sample of 316 participants is considerable within the constraints of Gaza, it is relatively small by large-scale intervention assessment standards [60]. Domain-level outcomes for concentration and sleep were also obtained from a subsection of participants as opposed to full cohorts, and only data immediately prior to and after the intervention was available, restricting conclusions about the program’s robustness long-term.

Additionally, while the qualitative data provides important contextual insights, this was based on interviews with participants who were all women and mothers’ given our desire not to place additional burden on staff by requesting specific interviews. As a result, there is a limited diversity of perspectives in the broader context of Gaza’s demographics [61]. Lastly, as client data was obtained retrospectively, pre- and post-treatment participant outcomes could not be compared to a control group, limiting the ability to infer a causal interaction between participation in Tarkiz and reduction in participants’ PTSD symptoms.

### 4.3 Conclusion

In conclusion, the evidence presented in this paper suggests that productive mental healthcare in contexts of harm should be modeled in a dynamic and adaptive way. By repositioning the ‘sick’ subject as the situation instead of the person and acknowledging a continuous context of harm in conflict-afflicted areas such as Gaza, it challenges existing therapeutic standards that necessitate stability as a precondition for healing. As more regions experience overlapping harms of war, displacement, disease and systematic violence, interventions such as Tarkiz invite practitioners and policymakers to expand the definition of healing to include circumstances where external stability is not possible, but resilience is.

## Supporting information

S1 Checklist Checklist. (DOCX)
